# Supplementary material for: Pollen assemblages and distribution characteristics in surface sediments of karst caves on the Guizhou Plateau, southwestern China
Source: PLoS One. 2025 Sep 22;20(9):e0333003. doi: 10.1371/journal.pone.0333003 (PMC12453262; doi:10.1371/journal.pone.0333003)
Supplement: S1 Table — (DOCX) [file pone.0333003.s001.docx]

**Pollen assemblages and distribution characteristics in surface sediments of karst caves on the Guizhou Plateau, southwestern China**

**Supporting information**

**S1 Table. Correlation coefficients for the pollen spectra from the samples at Yinhegong Cave** (significance levels *: P < 0.05; **: P < 0.01; ***: P < 0.001)

|  |  | **Ym** | **Y05** | **Y10** | **Y15** | **Y25** | **Y30** |
| --- | --- | --- | --- | --- | --- | --- | --- |
| **Ym** | **R** | 1.000 |  |  |  |  |  |
|  | **P-value** | — |  |  |  |  |  |
| **Y05** | **R** | 0.96*** | 1.000 |  |  |  |  |
|  | **P-value** | <0.0001 | — |  |  |  |  |
| **Y10** | **R** | 0.95*** | 1.00*** | 1.000 |  |  |  |
|  | **P-value** | <0.0001 | <0.0001 | — |  |  |  |
| **Y15** | **R** | 0.66*** | 0.83*** | 0.85*** | 1.000 |  |  |
|  | **P-value** | <0.0001 | <0.0001 | <0.0001 | — |  |  |
| **Y25** | **R** | 0.94*** | 0.99*** | 0.99*** | 0.87*** | 1.000 |  |
|  | **P-value** | <0.0001 | <0.0001 | <0.0001 | <0.0001 | — |  |
| **Y30** | **R** | 0.27 | 0.49** | 0.51*** | 0.86*** | 0.56*** | 1.000 |
|  | **P-value** | 0.118 | 0.002 | 0.001 | <0.0001 | 0.000 | — |
