# Supplementary material for: Pollen assemblages and distribution characteristics in surface sediments of karst caves on the Guizhou Plateau, southwestern China
Source: PLoS One. 2025 Sep 22;20(9):e0333003. doi: 10.1371/journal.pone.0333003 (PMC12453262; doi:10.1371/journal.pone.0333003)
Supplement: S2 Table — (DOCX) [file pone.0333003.s002.docx]

**Pollen assemblages and distribution characteristics in surface sediments of karst caves on the Guizhou Plateau, southwestern China**

**Supporting information**

**S2 Table. Correlation coefficients for the pollen spectra from the samples at Dongkou Cave** (significance levels *: P < 0.05; **: P < 0.01; ***: P < 0.001)

|  |  | **Dr** | **Dm** | **D05** | **D10** | **D15** | **D20** | **D25** | **D30** | **D35** | **D40** | **D45** | **Dc1** | **Dc2** |
| --- | --- | --- | --- | --- | --- | --- | --- | --- | --- | --- | --- | --- | --- | --- |
| **Dr** | **R** | 1.000 |  |  |  |  |  |  |  |  |  |  |  |  |
|  | **P-value** | — |  |  |  |  |  |  |  |  |  |  |  |  |
| **Dm** | **R** | 0.88*** | 1.000 |  |  |  |  |  |  |  |  |  |  |  |
|  | **P-value** | <0.0001 | — |  |  |  |  |  |  |  |  |  |  |  |
| **D05** | **R** | 0.99*** | 0.87*** | 1.000 |  |  |  |  |  |  |  |  |  |  |
|  | **P-value** | <0.0001 | <0.0001 | — |  |  |  |  |  |  |  |  |  |  |
| **D10** | **R** | 0.89*** | 0.87*** | 0.92*** | 1.000 |  |  |  |  |  |  |  |  |  |
|  | **P-value** | <0.0001 | <0.0001 | <0.0001 | — |  |  |  |  |  |  |  |  |  |
| **D15** | **R** | 0.77*** | 0.83*** | 0.80*** | 0.91*** | 1.000 |  |  |  |  |  |  |  |  |
|  | **P-value** | <0.0001 | <0.0001 | <0.0001 | <0.0001 | — |  |  |  |  |  |  |  |  |
| **D20** | **R** | 0.26 | 0.33* | 0.31* | 0.53*** | 0.57*** | 1.000 |  |  |  |  |  |  |  |
|  | **P-value** | 0.0740 | 0.0186 | 0.0262 | <0.0001 | <0.0001 | — |  |  |  |  |  |  |  |
| **D25** | **R** | 0.33*** | 0.46** | 0.37** | 0.59*** | 0.72*** | 0.86*** | 1.000 |  |  |  |  |  |  |
|  | **P-value** | 0.0182 | 0.0088 | 0.0088 | <0.0001 | <0.0001 | <0.0001 | — |  |  |  |  |  |  |
| **D30** | **R** | 0.49*** | 0.56*** | 0.52*** | 0.73*** | 0.88*** | 0.73*** | 0.86*** | 1.000 |  |  |  |  |  |
|  | **P-value** | 3.21E-4 | <0.0001 | <0.0001 | <0.0001 | <0.0001 | <0.0001 | <0.0001 | — |  |  |  |  |  |
| **D35** | **R** | 0.72*** | 0.70*** | 0.74*** | 0.83*** | 0.78*** | 0.52*** | 0.60*** | 0.77*** | 1.000 |  |  |  |  |
|  | **P-value** | <0.0001 | <0.0001 | <0.0001 | <0.0001 | <0.0001 | <0.0001 | <0.0001 | <0.0001 | — |  |  |  |  |
| **D40** | **R** | 0.76*** | 0.88*** | 0.77*** | 0.87*** | 0.95*** | 0.55*** | 0.76*** | 0.84*** | 0.73*** | 1.000 |  |  |  |
|  | **P-value** | <0.0001 | <0.0001 | <0.0001 | <0.0001 | <0.0001 | <0.0001 | <0.0001 | <0.0001 | <0.0001 | — |  |  |  |
| **D45** | **R** | 0.65*** | 0.60*** | 0.68*** | 0.66*** | 0.57*** | 0.36*** | 0.45*** | 0.47*** | 0.70*** | 0.62*** | 1.000 |  |  |
|  | **P-value** | <0.0001 | <0.0001 | <0.0001 | <0.0001 | <0.0001 | <0.0001 | <0.0001 | <0.0001 | <0.0001 | <0.0001 | — |  |  |
| **Dc1** | **R** | 0.44** | 0.49*** | 0.48*** | 0.40** | 0.40** | 0.18 | 0.23 | 0.23 | 0.28 | 0.50*** | 0.80*** | 1.000 |  |
|  | **P-value** | 0.00154 | 3.61E-4 | 4.98E-4 | 0.00411 | 0.00374 | 0.215 | 0.109 | 0.110 | 0.278 | 2.34E-4 | <0.0001 | — |  |
| **Dc2** | **R** | 0.18 | 0.18 | 0.21 | 0.23 | 0.19 | 0.31** | 0.31* | 0.16 | 0.24 | 0.26 | 0.80*** | 0.79*** | 1.000 |
|  | **P-value** | 0.223 | 0.200 | 0.149 | 0.110 | 0.197 | 0.0287 | 0.0275 | 0.255 | 0.0945 | 0.0655 | <0.0001 | <0.0001 | — |
