# Supplementary material for: Pollen assemblages and distribution characteristics in surface sediments of karst caves on the Guizhou Plateau, southwestern China
Source: PLoS One. 2025 Sep 22;20(9):e0333003. doi: 10.1371/journal.pone.0333003 (PMC12453262; doi:10.1371/journal.pone.0333003)
Supplement: Attachment — Submitted filename: Response to Reviewers.doc [file pone.0333003.s005.doc]

**Revision Notes**

**Editorial Board Member comments:**

Dear Dr. Tang,

Thank you for submitting your manuscript to PLOS ONE. After careful consideration, we feel that it has merit but does not fully meet PLOS ONE’s publication criteria as it currently stands. Therefore, we invite you to submit a revised version of the manuscript that addresses the points raised during the review process.

Please include the following items when submitting your revised manuscript:...

**[Authors’ response]** First of all, we would like to sincerely thank the Editorial Board Member for conducting this review on our manuscript. Thank you so very much for your support and time in evaluating our work. We greatly appreciate Dr. Tzen-Yuh Chiang and the anonymous reviewer for helpful comments and suggestions, all of which have been taken into account in our manuscript revisions, as detailed in our point-by-point response letter to reviewer. We also uploaded all files include 'Response to Reviewers', 'Revised Manuscript with Track Changes', and 'Manuscript'.

**Journal Requirements:**

1. Please ensure that your manuscript meets PLOS ONE's style requirements, including those for file naming.

**[Authors’ response]** Down.

2. In your manuscript, please provide additional information regarding the specimens used in your study. Ensure that you have reported human remain specimen numbers and complete repository information, including museum name and geographic location.

**[Authors’ response]** Not Applicable.

3. Thank you for stating the following financial disclosure:......

**[Authors’ response]** Thank you. Checked.

4. We note that your Data Availability Statement is currently as follows: “All relevant data are within the manuscript and in Supporting Information files.”

**[Authors’ response]** Yes. In revised version, We have compiled the relevant data in the supplementary materials.

5. We note that Figure 1 and 2 in your submission contain map/satellite images which may be copyrighted. All PLOS content is published under the Creative Commons Attribution License (CC BY 4.0), which means that the manuscript, images, and Supporting Information files will be freely available online, and any third party is permitted to access, download, copy, distribute, and use these materials in any way, even commercially, with proper attribution...

**[Authors’ response]** Thank you for your support. We have deleted the images (Fig 1C and D) from Google earth and added a data source explanation in the figure captions of Fig 1 and 2. Please see lines 97–100 and lines 128–132.

**Reviewers' comments:**

**Reviewer #1:** The topic of the article is interesting as it provides insights into how pollen reaching cave environments reflects surface vegetation. In this sense, the study partially meets its objective, offers valuable information on the subject, and is, in my opinion, of interest for publication. That said, the manuscript requires substantial revisions and further refinement before it can be considered for publication in PLOS One. Below are my comments for evaluation, consideration, and/or discussion by the editor and the authors. If there are any disagreements, please do not hesitate to reply or discuss them.

**[Authors’ response]** We would like to sincerely thank you for taking your valuable time to review our manuscript. All your comments and suggestions have been taken into account while revising our manuscript, and they have been very helpful for us to improve the quality of our manuscript.

**Main comments:**

1. Title: I believe the title does not fully reflect the results of the study, as the pollen assemblages are assessed but not interpreted within an environmental context or in terms of their significance. I suggest rephrasing it to better align with the study's discussion and findings.

**[Authors’ response]** We agree and your point is well taken. We have rephrasing it to: “Pollen assemblages and distribution characteristics in surface sediments of karst caves on the Guizhou Plateau, southwestern China” (Lines 1–2).

2. Materials and methods

-Sampling: It is unclear whether the same volume of sediment was sampled at each sampling point, which is essential for making valid comparisons. While the thickness of the sediment is mentioned, the surface area sampled is not specified. If this information cannot be provided, it may be difficult to make quantitative comparisons between samples of different volumes.

**[Authors’ response]** Sorry for the confusion. We have supplemented the information on the area of the surface sediment samples collected (10 cm×10 cm in area) in Lines 120–121 of the revised manuscript, in accordance with the actual situation of field sampling, to enhance the reproducibility of the sampling method.

3. Pollen analysis: Similar to the sampling concerns mentioned above, it is unclear what volume of sediment was initially extracted to obtain the 10 g of dried sample for further analysis. Clarifying this information would improve consistency and comparability between samples. I believe the methodology used for pollen analysis should be described in greater detail, particularly highlighting the key steps. Providing a clear and structured explanation of sample preparation, extraction techniques, chemical treatments, and identification criteria would enhance the study’s transparency and reproducibility. Additionally, specifying any quantification methods applied, would further strengthen the methodological clarity.

**[Authors’ response]** Thank you for your careful review. Yes, in fact, standards for sample preparation, extraction techniques, chemical treatments, and identification criteria have become very mature. We have revised this part to: approximately 8–15 mL of each dried sample was weighed. Please see line 141.

In addition, we have modified the Pollen analysis section in detail following your advice. Now, it changed to: The procedures are as follows: approximately 8–15 mL of each dried sample was weighed and transferred into a 500–mL plastic beaker. To remove carbonates, 50 mL of 15% hydrochloric acid (HCl) solution was added to each sample, followed by repeated rinsing with distilled water until neutral. Subsequently, 50 mL of 40% HF solution was introduced to dissolve silicates, after which the samples were again thoroughly washed again with distilled water to neutrality. The pollen residue was concentrated using a 7–µm nylon monofilament mesh in an ultrasonic bath to remove the tiny particles and then mounted in glycerin jelly for microscopic analysis. For quantitative pollen analysis, one tablet of Lycopodium spore (27,560 grains per tablet) was added to each sample as an exotic standard for calculating pollen concentration using the following formula:

$\text{Pollen concentration (grains/g)}=\frac{\text{L}\times\text{n}}{\text{l}\times\text{m}}$ (1)

Where *L* represents the number of Lycopodium spores added (27,560 grains), *l* denotes the number of Lycopodium spores counted, *n* is the pollen grains identified, and *m* refers to the sample weight (g or mL).

Please see lines 141–151. We hope that these changes/additions meet your expectation.

4. Results

-Table 1 should present the spore-pollen types for each cave separately, rather than in the current format. As it stands, it is difficult to determine which types are found in each cave. Given that the authors discuss both caves separately, as they should, I recommend restructuring the table to emphasise this distinction more clearly.

**[Authors’ response]** Your point is well taken. We have reorganized Table 1 (Lines 169–170) to clearly show the differences in pollen and spore types between the two caves.

-Correlation analysis: It is unclear which dataset was used for the correlation analysis. To ensure reproducibility and reusability, I recommend providing the dataset as a supplementary file (the specific pollen counts being compared should be explicitly stated for a meaningful interpretation). Furthermore, the statistical significance of the correlation (p-value) is not reported. To ensure a complete statistical interpretation, both R and p-values should be provided.

**[Authors’ response]** Sorry for the confusion. We have performed an updated correlation analysis, with full results (including p-values) now available in Supplementary s 1 and 2 Tables. In addition, we have incorporated significance level annotations in Figure 6 to enhance statistical clarity. Please see Line 221, Fig 6, S1 and S2 Tables. The dataset used for the correlation analysis has been organized in the Supporting Information. Please see Line 219, S1 and S2 Datasets.

5. Discussions and Conclusions

-Section: Cave environmental humidity – The relative humidity measured at each sampling point should be included in your assessment. As it stands, this section is general and primarily based on common knowledge that cave humidity is typically high. However, you do not provide any specific humidity measurements or discuss its variation in your results. Including this data would strengthen the analysis and help contextualise its potential influence on pollen preservation and deposition.

**[Authors’ response]** Thank you for your careful review. We agree. As one of the influence factors of surface sediment pollen assemblages in caves, environmental humidity may lead to significant spatial heterogeneity^[1]^. Quantitative measurement is one method to solve this problem. In this study, although we did not measurement cave humidity, previous research results was used to qualitatively indicate cave humidity^[2-5]^. Davis noted that dry cave environments, such as those found in Bechan in the western United States, are conducive to pollen preservation, while humid conditions tend to promote microbial activity, which accelerates pollen decay and makes the environment unfavorable for pollen preservation^[2]^. Thus, after careful consideration, we have added references 40 and 41 and modified the Discussion. Please see lines 302–308.

References:

1. Hunt CO, Fiacconi M. Pollen taphonomy of cave sediments: What does the pollen record in caves tell us about external environments and how do we assess its reliability ?. Quat Int. 2018; 485, 68–75.

2. Davis OK. Caves as sources of biotic remains in arid western North America. Palaeogeogr Palaeoclimatol Palaeoecol 1990; 76 (3–4): 331–348.

3. Navarro C, Carrión JS, Prieto AR, Munuera M. Modern cave pollen in an arid environment and its application to describe palaeorecords. Complutum. 2002; 13, 7–18.

4. Navarro C, Carrión J S, Munuera M, Prieto AR. Cave surface pollen and the palynological potential of karstic cave sediments in palaeoecology. Rev Palaeobot Palynol. 2001; 117, (4): 245‒265.

5. Navarro Camacho C, Carrión JS, Navarro J, Munuera M, Prieto AR. An experimental approach to the palynology of cave deposits. J Quat Sci. 2000; 15, (6): 603–619.

40. Yin C, Zhou Z, Cao M, Zhang J, Zhang Q, Zhang S, et al. Analysis on the relationship between climatic and environmental factors in karst caves: An example from Zhijin Cave of Guizhou Province. Carsologica sinica. 2016; 35 (4): 414–424. Chinese

41. Shi L, Zhou Z, Fan B, Tang Y, Yan L, An D, et al. Characteristics of Ventilation Effect in Karst Caves and Its Influence on Cave Air Environment: A Case Study of Mahuang Cave of Suiyang, Guizhou Province. Resour Environ Yangtze Basin. 2021; 30 (7): 1704–1713. Chinese

6. General comment:

The authors discuss factors influencing forest cover around the caves and its impact on pollen counts; however, these aspects are only vaguely mentioned, despite their crucial role in shaping the observed patterns. Forest cover can be easily inferred from existing maps using Land Use/Land Cover Classification (GIS & Spatial Analysis), a relatively simple and time-efficient approach that would enhance the study's interpretation. Alternatively, models estimating vegetation cover based on modern pollen deposition patterns in similar environments could provide valuable insights. Historical land cover data, aerial imagery, and satellite observations could also be used to compare present-day vegetation with past trends. Additionally, integrating environmental and climatic data, such as temperature, precipitation, and topographic features, would help contextualise vegetation distribution. To improve the robustness of the analysis, these factors should be incorporated into multifactorial analyses, either as quantitative or qualitative variables, to better assess the relationship between surface and subsurface pollen assemblages and their environmental drivers. If none of these approaches are feasible, the authors should explicitly acknowledge this limitation and discuss its potential impact on their conclusions.

**[Authors’ response]** Thank you for your comment. We agree. Land Use/Land Cover Classification (GIS & Spatial Analysis) was one of the easily methods to obtain the forest cover. However, its spatial resolution changed from 10 to 30 m. In this study, the area is very small. The impact of forest cover on the sedimentation inside the cave does not involve large-scale Land Use/Land Cover data.Thus, in this study, we conducted in-depth field investigations on the vegetation outside the cave and provided detailed descriptions in the revised version. Moreover, to incorporate your suggestion, we also add some background information in the “Description of the caves” section. Please see 2 Description of the caves. We hope that these changes/additions meet your expectation.

**Minor comments and questions**

1. Abstract

-Lines 14-15: The rationale for selecting these two specific caves remains unclear within the context provided by the authors.

**[Authors’ response]** Thank you for catching this. To address this, we have modified this sentence to: Cave pollen has great potential applications in reconstruction of past environments; However, the representativeness of cave pollen to the external environment remains controversial, especially in complex cave systems. This study conducted pollen analysis in surface sediment samples from two karst caves with complex geometry on the Guizhou Plateau. Please see lines 12–15.

2. Introduction

-Line 33: Providing a specific timespan would help clarify the temporal resolution of pollen analysis in reconstructing palaeovegetation or palaeoclimate.

**[Authors’ response]** Modified. Now this sentence changed to：Fossil pollen preserved in these sediments has been used successfully in some case studies to reconstruct Quaternary paleovegetation and paleoclimate, and the method has shown great potential [6–13]. Please see lines 33–34.

3. Line 48: Could you please elaborate on what specific subsequent research you or the referenced authors are referring to ? Providing more context or examples would help clarify how this study contributes to or aligns with future research directions.

**[Authors’ response]** Sorry for the confusion. We have deleted this sentence after carefully consideration. Please see line 49.

4. Lines 74-76: The connection between the study’s aims and the archaeological records or past human occupation is unclear. As currently presented, these lines do not provide relevant context for the research focus and should be removed unless further justification is provided.

**[Authors’ response]** Thanks. We have removed it as suggested. Please see lines 74–76.

5. Lines 94-98, 112-115: Relevant references should be included to support these statements. If these descriptions are based on the authors’ own evaluations, they should be moved to the Results section rather than remaining in the introduction.

**[Authors’ response]** Thank you for catching this. We have added the reference in the text (Lines 92–96, Lines 111–114).

6. Results

-Line 202: The mention of surface sediment should be removed here, as it was already discussed in the previous paragraph. The current paragraph appears to focus on mosses, and keeping surface sediment in this context may cause confusion.

**[Authors’ response]** We apologize for the confusion caused by our previous wording. We have now corrected the term “surface sediment” to “surface soil.” Section 4.2 mainly presents the results of pollen analysis from both the inside and outside of the Dongkou Cave. The first paragraph describes the pollen analysis results from the cave interior’s surface sediment, while the second paragraph provides an overview of the pollen analysis results from the two types of surface samples collected outside the cave, namely surface soil sample and fresh moss sample. As we have already mentioned in the sample collection section, two different types of surface samples were collected outside the cave, including one surface soil sample and one fresh moss sample. Therefore, the second paragraph offers a comprehensive introduction to the pollen analysis results of these two samples. Please see line 210.

7. Discussion

-Line 225: Please clarify what the consistent pattern refers to or is in relation to. The statement lacks clarity and should specify the context or comparison being made.

**[Authors’ response]** Sorry for the confusion. The statement "consistent pattern" here refers to the similarity between the cave surface pollen assemblages and the external pollen rain. We have revised the relevant statements to make them clearer. Please see lines 233–236.

8. Lines 249-251: These statements belong to the Results section and should be moved accordingly for better structure and clarity.

**[Authors’ response]** Agree. We have integrated these statements into the Result section (Lines 219–227) as suggested.

9. Lines 274-276: On what grounds is this statement based ? Please provide more specificity, supporting data, or a relevant reference to substantiate the claim.

**[Authors’ response]** We apologize for the confusion. We have deleted this statement due to less supporting data. Please see line 283.

10. Lines 334-338: The phenomenon described, specific to caves modified for tourism or show caves, is known as "lampenflora". Please expand on this concept in your discussion and include relevant comparisons with existing literature that examines "lampenflora", its ecological implications, and how it relates to the findings of your study.

**[Authors’ response]** Thank you for the excellent suggestion. We have incorporated the concept of "lampenflora" into the revised version and expanded the discussion accordingly (Lines 346–350).

11. Lines 351-357: It would be valuable to elaborate further on how vegetation from different distances can be represented in pollen assemblages found inside caves. Are there estimates on the spatial scale of vegetation input based on existing studies? Vegetation maps detailing areas at varying proximities to the caves are likely available, and incorporating such data would enhance the study by providing an estimate of the pollen source area. This would be particularly useful for researchers working in this field, helping them contextualise similar findings in different settings.

**[Authors’ response]** Thank you for your suggestion. We have added related studies in in the revised version. Please see lines Please see lines 363–372.

12. Conclusions

-Line 367: It is unclear why the middle-to-rear parts of caves should be treated with caution. Could you clarify in relation to what? Are you referring to pollen deposition patterns, post-depositional processes, or other environmental factors affecting pollen preservation? Providing a more detailed explanation would help readers better understand the reasoning behind this statement.

**[Authors’ response]** We apologize for the confusion. We have deleted this conclusion due to less supporting data.

Thank you for your comment again. Your comments are valid and challenging. To incorporate your points, we also checked our manuscript carefully. We believe the changes/additions made are a substantial improvement and hope that they meet your expectation.
