# Supplementary material for: Pollen assemblages and distribution characteristics in surface sediments of karst caves on the Guizhou Plateau, southwestern China
Source: PLoS One. 2025 Sep 22;20(9):e0333003. doi: 10.1371/journal.pone.0333003 (PMC12453262; doi:10.1371/journal.pone.0333003)
Supplement: Attachment — Submitted filename: Response to Reviewers.docx [file pone.0333003.s006.docx]

**6. Review Comments to the Author**

**Reviewer #1:**

The authors have made a great effort to improve the manuscript and to address the issues raised during the review process. In my opinion, the work is now suitable for publication.

**[Authors’ response]**

We sincerely appreciate your hard work and valuable comments on our manuscript; we are very grateful to hear that our revisions are acceptable.

**Reviewer #2:**

1. There are some grammar errors in English expression in some places (such as adding 'a' before 'great' in the first sentence of the Abstract). It is recommended to have an English professional polish it;

**[Authors’ response]**

Thank you for your careful review. We have implemented your suggestions (Line 12) and conducted a comprehensive revision of the manuscript; the editing certificate has been uploaded to the peer review system.

2. In the Instruction, it is necessary to supplement the research on the paleoclimatic records of stalagmites, as it is an important geological historical archive material in karst areas;

**[Authors’ response]**

Thank you for your careful review and constructive suggestions. Yes, stalagmite record is an important geological historical archive material in karst areas. This paper focuses on pollen assemblages and distribution characteristics in surface sediments of karst caves.

3. The Line 32-34 statement indicates that since the paleovegetation and paleoclimate have been successfully reconstructed using pollen, the existing problems have been solved. Does this contradict the following problem description?

**[Authors’ response]**

Thank you very much for pointing out the contradictory expressions in the later sections; We have made careful revisions accordingly. Please see Lines 33–34.

4. Suggest removing the reference "7" from Line 55, as it is a study from over 20 years ago and should not be classified as a "current research";

**[Authors’ response]**

Thank you. We have deleted the reference “7” in the text as suggested (Line 55).

5. The starting point for the problem of multi-channel and multi cave is not the best. I suggest starting from scientific problems, that is, what kind of problems need to be solved through this study in the process of reconstructing paleoclimate and paleoenvironment using cave sediment pollen;

**[Authors’ response]**

Thank you for your kind advise. In fact, current studies are mostly focused on simple caves with a single entrance and a single passage. However, research on more complex caves with multiple entrances, passages, and chambers remains very limited. Thus, the purpose of the study was to reveal the indicative role of complex cave surface pollen assemblages in reflecting external vegetation and to explore the potential factors influencing cave pollen taphonomic processes. Please see changes in “Abstract”.

6. The description of the opening on Line 89-90 is inaccurate, with a width of 8 m and a height of 4.5 m. How could it be nearly circular ?

**[Authors’ response]**

Thank you for catching this. We have corrected the cave entrance morphology to an elliptical shape. Please see Lines 86–87.

7. What is the propagation distance of pollen in the research area, and what are the wind speed and direction? What is the scope of this research's investigation ? These pieces of information need to be described clearly (Line 94-95);

**[Authors’ response]**

Thank you for your comments. In fact, the relationship between propagation distance of pollen and instantaneous wind is still challenging to study. However, palynologists have conducted studies on the relationship between modern cave pollen assemblages and external pollen rain and vegetation composition. In this study, we have modified Figures 1 and 2 to show the study scope in detail.

8. Why is the complex cave feature repeatedly emphasized by the author not reflected in Line 234?

**[Authors’ response]**

We apologize for the confusion. At the beginning of section 5.1, we have already reaffirmed the structure of the two complex cave systems, as shown in Lines 232–234—Our pollen analysis of complex caves with various geometry indicated that in both the multi-entrance multi-passage Yinhegong Cave and the single-entrance multi-chamber Dongkou Cave with significant variations in passage width, ….

9. In Line 242, the specific distance may be related to the type and intensity of ventilation. How did the authors consider these factors?

**[Authors’ response]**

Thank you for your careful comments. In fact, the relationship between propagation distance of pollen and instantaneous wind is still challenging to study. In complex caves, instantaneous wind speed changes constantly. In the present study, in both the multi-entrance, multi-passage Yinhegong Cave and the single-entrance, multi-chamber Dongkou Cave, with significant passage width variation, pollen assemblage in the surface sediment within 5–15 m of the entrance exhibited strong correspondence with external pollen rain, effectively reflecting the composition of the external vegetation. Our study can only reflect the existence of this phenomenon and new finds. The formation mechanism needs to be analyzed further with long-term monitoring.

10. Is a distance of tens of meters sufficient (Line 354)? What is the basis? How representative is it?

**[Authors’ response]**

Thank you. We designed this study to collect surface soil and fresh moss samples within tens of meters around the cave entrance for modern pollen rain analysis. This experimental approach was adopted because it remains unclear whether pollen assemblages in cave surface sediments correspond to external vegetation composition. Even if such correspondence exists, the spatial range over which these pollen assemblages can accurately reflect external vegetation characteristics is also uncertain in our study area. Thus, this experiment serves as an exploratory investigation to address these uncertainties.

The rationale for our experimental design is twofold. First, it is based on the definitions of local and regional spatial scales outside the cave provided by Coles et al. (1898). Second, we drew on the spatial distribution ranges of modern sample collection points used in previous studies, such as those by Navarro et al. (2001, 2002), de Porras et al. (2011), Fiacconi and Hunt (2017), and Yang et al. (2021). The studies typically confined their sampling to within a 200-m radius around the cave entrance. Our decision to focus on a smaller radius (tens of meters) around the cave entrance is intended to provide a more detailed and localized understanding of the relationship between cave pollen assemblages and external vegetation.

Our study reveals that the surface pollen assemblages within the 5–15 m zone at the front of the cave entrance exhibit good correspondence with the external vegetation. This finding is consistent with the research of de Porras et al. (2011), Fiacconi and Hunt (2015), and Yang et al. (2021) However, the relationship between the pollen assemblages and vegetation at greater spatial distances remains unclear. We have explicitly acknowledged this limitation in our discussion (Lines 366–371) and suggest that future research should address this gap by increasing the collection of modern samples and conducting vegetation surveys across different spatial ranges outside the cave. This approach will help to better define the spatial representativeness of cave surface pollen assemblages.

*References:*

*Coles GM, Gilbertson DD, Hunt CO, Jenkinson RDS. Taphonomy and the palynology of cave deposits. Cave Sci. 1989; 16 (3): 83–89.*

*Navarro C, Carrión JS, Munuera M, Prieto AR. Cave surface pollen and the palynological potential of karstic cave sediments in palaeoecology. Rev Palaeobot Palynol. 2001; 117, (4): 245‒265.*

*Navarro C, Carrión JS, Prieto AR, Munuera M. Modern cave pollen in an arid environment and its application to describe palaeorecords. Complutum. 2002; 13, 7–18.*

*de Porras ME, Mancini MV, Prieto AR. Modern pollen analysis in caves at the Patagonian steppe, Argentina. Rev Palaeobot Palynol. 2011; 166, 335‒343.*

*Fiacconi M, Hunt CO. Pollen taphonomy at Shanidar Cave (Kurdish Iraq): An initial evaluation. Rev Palaeobot Palynol. 2015; 223, 87–93.*

*Fiacconi M, Hunt CO. Palynology of surface sediments from caves in the Zagros Mountains (Kurdish Iraq): Patterns and processes. Rev Palaeobot Palynol. 2017; 239, 66–76.*

*Yang Q, Zhao K, Zhou X, Wang J, Chen G, Li D, et al. Evaluation of the potential of surface pollen spectra from caves in SW China for vegetation reconstruction. Quat Int. 2021; 591, 119–128.*

**Reviewer #3:**

This is an interesting paper on cave pollen study. However, after I reviewed the revised manuscript, I do not think the authors took it seriously. The following concerns should be solved before publication.

1. There are several grammatical errors or typos. E.g.,

Line 39, ‘…but is also the key…’ should be ‘…, but also the key…’

Line 132, ‘using a DJI drone,)’, the comma after the word ‘drone’ should be deleted.

Line 144, ‘…after which the samples were again thoroughly washed again with…’ the first ‘again’ is a repeated word.

Line 233, ‘Our pollen analysis of complex caves with various geometry indicated that,, in both the multi-entrance’, the comma after the word ‘that’ should be deleted.

Line 236, ‘…pattern similar to that of the external pollen rain, albeit with minor differences (Fig 4–6).’ (Fig 4–6) should be ‘(Figs 4–6).’

**[Authors’ response]**

Thank you for your careful review. We have corrected the above grammatical errors and typos according to your suggestions.

2. There are three wrong Latin names in Table 1. Anacaodiaceae should be ‘Anacardiaceae’, Aluns should be ‘Alnus’, and Vsitis should be ‘Vitis’. Also Line 172, Aluns should be ‘Alnus’

**[Authors’ response]**

Thank you for catching these. We have corrected the wrong Latin names in Table 1, Figures 4 and 5, and S1 and S2 Datasets, and updated the figures.

3. I would like to see all the pollen and spore pictures. You said you found 69 taxa, then you should put all of them in the Fig.3

**[Authors’ response]**

Thank you very much for your suggestion. Since the focus of our study is not on pollen morphology, we did not take photos of all the pollen types during the identification. Consequently, we apologize that we cannot provide photos of the 69 pollen taxa mentioned in this paper. To avoid inconsistency between the text and the figure, we have revised the caption of Figure 3 accordingly (Line 170).

4. On the 4.3 Correlation analysis, I can not follow the numbers you stated. Lines 220-221, ‘…ranged from 0.18 to 0.98’, I cannot find the corresponding figures both in fig 6 and S1 and S2 tables. Plus, the significance levels are different. You use significance levels *P < 0.05, **P < 0.01, ***P < 0.001 in S1 and S2 Tables, but they are P<0.0001 in the table text.

**[Authors’ response]**

We apologize for the confusion. Thank you very much for pointing out this issue. We have carefully checked the original results and found that the data in Figure 6 were misaligned. Additionally, the R values in SI and S2 Tables had not been rounded off, whereas the R values shown in Figure 6 had indeed been rounded off. Therefore, we have revised and updated the Figure 6 and SI and S2 Tables to ensure consistency in the results.

The range of the correlation coefficients (R) has been revised to 0.18–0.99, with the specific results highlighted using yellow and red rectangular boxes in Figure 6.

**Fig 6. Correlation coefficients for pollen spectra from the samples of Yinhegong Cave (A) and Dongkou (B) Cave** (significance levels *: P < 0.05; **: P < 0.01; ***: P < 0.001)

In SI and S2 Tables, the values in rows labeled "P" (for example, the values highlighted with a yellow background in SI Table) represent the specific P-values at different significance levels (significance levels P < 0.05, P < 0.01, and P < 0.001); that is, the probability of the observed sample data occurring. To avoid confusion, we have revised the labels "P" to "P-value" in SI and S2 Tables.

**S1 Table. Correlation coefficients for the pollen spectra from the samples at Yinhegong Cave** (significance levels * P < 0.05, ** P < 0.01, *** P < 0.001)

|  |  | **Ym** | **Y05** | **Y10** | **Y15** | **Y25** | **Y30** |
| --- | --- | --- | --- | --- | --- | --- | --- |
| **Ym** | **R** | 1.000 |  |  |  |  |  |
|  | **P** | — |  |  |  |  |  |
| **Y05** | **R** | 0.96*** | 1.000 |  |  |  |  |
|  | **P** | <0.0001 | — |  |  |  |  |
| **Y10** | **R** | 0.95*** | 1.00*** | 1.000 |  |  |  |
|  | **P** | <0.0001 | <0.0001 | — |  |  |  |
| **Y15** | **R** | 0.66*** | 0.83*** | 0.85*** | 1.000 |  |  |
|  | **P** | <0.0001 | <0.0001 | <0.0001 | — |  |  |
| **Y25** | **R** | 0.94*** | 0.99*** | 0.99*** | 0.87*** | 1.000 |  |
|  | **P** | <0.0001 | <0.0001 | <0.0001 | <0.0001 | — |  |
| **Y30** | **R** | 0.27 | 0.49** | 0.51*** | 0.86*** | 0.56*** | 1.000 |
|  | **P** | 0.118 | 0.002 | 0.001 | <0.0001 | 0.000 | — |

S1 and S2 Tables have been revised to the following format.

Thank you for your comment again. Your comments are valid and challenging. To incorporate your points, we also checked our manuscript carefully. We believe the changes/additions made have improved the quality of the paper substantially, and we hope that they meet your expectations.
