# Supplementary material for: Pollen assemblages and distribution characteristics in surface sediments of karst caves on the Guizhou Plateau, southwestern China
Source: PLoS One. 2025 Sep 22;20(9):e0333003. doi: 10.1371/journal.pone.0333003 (PMC12453262; doi:10.1371/journal.pone.0333003)
Supplement: Attachment — Submitted filename: Response_to_Reviewers_auresp_3.doc [file pone.0333003.s007.doc]

**Revision Notes**

**Editorial Board Member comments:**

Dear Dr. Tang,

Thank you for submitting your manuscript to PLOS ONE. After careful consideration, we feel that it has merit but does not fully meet PLOS ONE’s publication criteria as it currently stands. Therefore, we invite you to submit a revised version of the manuscript that addresses the points raised during the review process.

Please submit your revised manuscript by Aug 29 2025 11:59PM. If you will need more time than this to complete your revisions, please reply to this message or contact the journal office at [plosone@plos.org](mailto:plosone@plos.org). When you're ready to submit your revision, log on to https://www.editorialmanager.com/pone/ and select the 'Submissions Needing Revision' folder to locate your manuscript file.

Please include the following items when submitting your revised manuscript: ...

**[Authors’ response]**

We would like to sincerely thank the Editorial Board Member for conducting this review on our manuscript. Thank you so very much for your support and time in evaluating our work. We greatly appreciate Dr. Tzen-Yuh Chiang and the anonymous reviewer for helpful comments and suggestions, all of which have been taken into account in our manuscript revisions, as detailed in our point-by-point response letter to reviewers. We also uploaded all files include 'Response to Reviewers', 'Revised Manuscript with Track Changes', and 'Manuscript'.

**Journal Requirements:**

If the reviewer comments include a recommendation to cite specific previously published works, please review and evaluate these publications to determine whether they are relevant and should be cited. There is no requirement to cite these works unless the editor has indicated otherwise.

**[Authors’ response]**

Thank you for the reminder. We have carefully examined the reviewer comments and confirm that no specific publications were recommended for citation. Accordingly, no additional references have been added.

**Reviewers' comments:**

**6. Review Comments to the Author**

Please use the space provided to explain your answers to the questions above. You may also include additional comments for the author, including concerns about dual publication, research ethics, or publication ethics. (Please upload your review as an attachment if it exceeds 20,000 characters)

**Reviewer #1:** The authors have carefully considered the reviewers’ comments and addressed them where appropriate, while remaining aligned with the original scope and objectives of their study. The revisions have improved the clarity and presentation of the manuscript. In my view, the work is now suitable for publication.

**[Authors’ response]**

We sincerely appreciate your constructive feedback and are pleased that our revisions have addressed your concerns effectively. Your insightful suggestions significantly improved the manuscript’s clarity and scholarly rigor. We are grateful for your time and expertise in evaluating our work and are honored by your endorsement for publication.

**Reviewer #2:** This manuscript has passed two rounds of expert reviews and revisions, but I believe that overall there has been no substantial improvement, especially in answering most of the questions raised by the reviewers, which were not fully adopted in the manuscript revision and did not provide convincing reasons for rejecting the revisions. Include discussions on paleoclimate records such as stalagmites and scientific questions (research objectives) in the introduction section. And many contradictory statements still exist in many places. In addition, there is no necessary description of the cave sediments mentioned in the manuscript, and it is difficult to determine whether they are suitable as archives for paleoclimate reconstruction (at least dating and sedimentary sequences must be regular). Therefore, I have to suggest rejection.

**[Authors’ response]**

We sincerely thank you for your constructive comments. Nevertheless, we regret that our previous revision did not fully meet your expectations, and we are truly sorry for this shortcoming. Consequently, we have carefully re-examined the two rounds of feedback provided by all three reviewers, paying particular attention to the issues you raised in both your previous and the present comments. On this basis, we have undertaken another thorough revision, hoping that the changes will further improve the quality of the manuscript.

Below, we address the three specific points raised in your latest comments and detail the corresponding modifications and clarifications.

(1) Include discussions on paleoclimate records such as stalagmites and scientific questions (research objectives) in the introduction section.

**[Authors’ response]**

We sincerely thank you for your careful review and constructive comments. While we fully acknowledge that speleothems—particularly stalagmites—offer outstanding advantages in palaeoclimate reconstruction because of their high-resolution geochemical archives, this paper is explicitly devoted to the relationship between modern cave pollen assemblages and the external vegetation and environment. This modern process investigation is designed to serve as an analogue for clarifying how fossil pollen preserved in cave sediments reflects contemporaneous external conditions.

Accordingly, we have refrained from providing an in-depth review or critical assessment of stalagmite-based palaeoclimate research in the Introduction. Instead, we focus on clearly articulating the objectives and significance of the modern cave pollen study (Please see Lines: 36–43). Furthermore, a survey of the international literature specifically focused on modern cave pollen analysis (*e.g., Fiacconi and Hunt, 2015, 2017; Hunt and Fiacconi, 2018; Navarro et al., 2001, 2012; Yang et al., 2021*) reveals that these papers seldom introduces stalagmites or their palaeoclimate applications in either the Introduction or the Discussion. For these reasons, we believe that a discussion of stalagmite-based palaeoclimate research is not essential to this paper, and we therefore prefer not to add such content.

In this revised version, we have further clarified the scientific questions and refined the corresponding statements— ... whether fossil pollen preserved in cave sediments faithfully reflects contemporaneous environmental information beyond the cave [5, 14–18]. To reduce these uncertainties, research into modern cave pollen processes is indispensable, as it provides the critical modern analogue for interpreting the taphonomy and environmental significance of such archives [14, 19, 20]—in the Introduction. Please see Lines 36–40.

***References:***

*Fiacconi M, Hunt CO. Pollen taphonomy at Shanidar Cave (Kurdish Iraq): An initial evaluation. Rev Palaeobot Palynol. 2015; 223, 87–93.*

*Fiacconi M, Hunt CO. Palynology of surface sediments from caves in the Zagros Mountains (Kurdish Iraq): Patterns and processes. Rev Palaeobot Palynol. 2017; 239, 66–76.*

*Hunt CO, Fiacconi M. Pollen taphonomy of cave sediments: What does the pollen record in caves tell us about external environments and how do we assess its reliability ?. Quat Int. 2018; 485, 68–75.*

*Navarro C, Carrión JS, Munuera M, Prieto AR. Cave surface pollen and the palynological potential of karstic cave sediments in palaeoecology. Rev Palaeobot Palynol. 2001; 117, (4): 245‒265.*

*Navarro C, Carrión JS, Prieto AR, Munuera M. Modern cave pollen in an arid environment and its application to describe palaeorecords. Complutum. 2002; 13, 7–18.*

*Yang Q, Zhao K, Zhou X, Wang J, Chen G, Li D, et al. Evaluation of the potential of surface pollen spectra from caves in SW China for vegetation reconstruction. Quat Int. 2021; 591, 119–128.*

(2) And many contradictory statements still exist in many places.

**[Authors’ response]**

Thank you for your meticulous and responsible review, which brought to light several contradictory statements in our manuscript. We have thoroughly re-examined the text and have made the following revisions accordingly.

We have revised “Fern spores were mainly represented by Gleicheniaceae (5.0%) and Polypodiaceae (3.5%), with contents decreasing toward the interior of the cave (Fig 5B)” to “Fern spores were mainly represented by Gleicheniaceae (5.0%) and Polypodiaceae (3.5%), showing higher percentages in both the entrance and rear sections (Fig 5B)”. (Lines 203–204)

The contradictory statement noted in the previous round review—Fossil pollen preserved in these sediments has been used successfully in some case studies to reconstruct Quaternary paleovegetation and paleoclimate, and the method has shown great potential—was removed during the last revision and has been replaced with the following wording: Fossil pollen sequences preserved in these sediments hold great potential for reconstructing Quaternary paleovegetation and paleoclimate changes [6–13]. (Lines 31–34)

(3) In addition, there is no necessary description of the cave sediments mentioned in the manuscript, and it is difficult to determine whether they are suitable as archives for paleoclimate reconstruction (at least dating and sedimentary sequences must be regular).

**[Authors’ response]**

We sincerely thank you for your professional suggestions from the perspectives of sedimentology and geochronology. Indeed, the establishment of a high-precision, reliable chronological framework and the detailed description of stratigraphic sequences—including lithology and stratigraphic contact—constitute the indispensable foundation for any robust paleoenvironmental reconstruction.

This study focuses primarily on the relationship between modern cave pollen and external vegetation—in other words, on modern pollen processes within the cave—rather than on paleoclimate reconstruction based on cave sedimentary strata. Consequently, the samples collected here consist only of the uppermost ~1 cm surface layer on the cave floor (see Materials and Methods, lines 117–118), not a deep stratigraphic profile. Therefore, chronological dating and descriptions of lithology and stratigraphic contacts are not required.

In addition, we have corrected the spelling errors in Figures 4 and 5 and updated the figures accordingly. For all other detailed revisions, please refer to “Revised Manuscript with Track Changes”.

Thank you for your comments again. Your comments are valid and challenging. To incorporate your points, we also checked our manuscript carefully. We believe the changes/additions made have improved the quality of the paper substantially, and we hope that they meet your expectations.
