# Supplementary material for: Pollen assemblages and distribution characteristics in surface sediments of karst caves on the Guizhou Plateau, southwestern China
Source: PLoS One. 2025 Sep 22;20(9):e0333003. doi: 10.1371/journal.pone.0333003 (PMC12453262; doi:10.1371/journal.pone.0333003)
Supplement: Attachment — Submitted filename: Response_to_Reviewers_auresp_4.doc [file pone.0333003.s008.doc]

**Revision Notes**

**Editorial Board Member comments:**

Dear Dr. Tang,

Thank you for submitting your manuscript to PLOS ONE. After careful consideration, we feel that it has merit but does not fully meet PLOS ONE’s publication criteria as it currently stands. Therefore, we invite you to submit a revised version of the manuscript that addresses the points raised during the review process.

Please submit your revised manuscript by Sep 27 2025 11:59PM. If you will need more time than this to complete your revisions, please reply to this message or contact the journal office at [plosone@plos.org](mailto:plosone@plos.org). When you're ready to submit your revision, log on to https://www.editorialmanager.com/pone/ and select the 'Submissions Needing Revision' folder to locate your manuscript file.

Please include the following items when submitting your revised manuscript: ...

**[Authors’ response]**

We would like to sincerely thank the Editorial Board Member for conducting this review on our manuscript. Thank you so very much for your support and time in evaluating our work. We greatly appreciate Dr. Tzen-Yuh Chiang and the anonymous reviewers for helpful comments and suggestions, all of which have been taken into account in our manuscript revisions, as detailed in our point-by-point response letter to reviewers. We also uploaded all files include 'Response to Reviewers', 'Revised Manuscript with Track Changes', and 'Manuscript'.

**Journal Requirements:**

If the reviewer comments include a recommendation to cite specific previously published works, please review and evaluate these publications to determine whether they are relevant and should be cited. There is no requirement to cite these works unless the editor has indicated otherwise.

Please review your reference list to ensure that it is complete and correct. If you have cited papers that have been retracted, please include the rationale for doing so in the manuscript text, or remove these references and replace them with relevant current references. Any changes to the reference list should be mentioned in the rebuttal letter that accompanies your revised manuscript. If you need to cite a retracted article, indicate the article’s retracted status in the References list and also include a citation and full reference for the retraction notice.

**[Authors’ response]**

Thank you for this helpful guidance. We have carefully reviewed the reviewer’s suggestions and examined every reference in our list. No additional citations were recommended beyond those already included, and we confirm that none of the works we cite have been retracted. Consequently, the reference list remains unchanged.

**Reviewers' comments:**

**6. Review Comments to the Author**

Please use the space provided to explain your answers to the questions above. You may also include additional comments for the author, including concerns about dual publication, research ethics, or publication ethics. (Please upload your review as an attachment if it exceeds 20,000 characters)

**Reviewer #1:** I stand by my decision that the authors have carefully considered the reviewers’ comments and addressed them where appropriate, while remaining aligned with the original scope and objectives of their study. The revisions have improved the clarity and overall presentation of the manuscript.

In my view, the work is suitable for publication. The authors have remained focused on the topic as specified in the title and, as agreed in the previous revision, have removed sections not directly related to it. They have strengthened the relevant sections using appropriate methodology, clearly presented the results, and discussed them in their proper context.

**[Authors’ response]**

Thank you very much for your thorough review and for confirming that our revisions have adequately addressed all comments while remaining faithful to the original scope of the study. We appreciate your positive assessment of the improved clarity and presentation, as well as your endorsement of the manuscript’s suitability for publication. Your guidance has been invaluable in helping us strengthen the work, and we are grateful for your continued support.

**Reviewer #4:** Although cave sediment is not ideal for reconstructing past environments and climate, it holds significant importance for studying ancient humans and their habitats. I recommend the authors refine their presentation of this point in the Abstract and Introduction.

**[Authors’ response]**

We are truly grateful for your constructive feedback. We agree that cave sediments are irreplaceable for understanding ancient humans and their habitats. In response, we have revised the Abstract to foreground the archaeological significance of our record (lines 12–14). The Introduction already contains an extended discussion of the same issue (Please see Lines 62–71); no further changes were necessary there.

**Reviewer #5:** The manuscript reports an interesting and important study on surface pollen in karst caves. Cave sediments in some regions, especially regions without good and long-enough lake sediments or continuous aeolian deposits, are good records for paleoenvironment reconstruction. I am glad to see the efforts paid by these authors trying to understand the basics of pollen studies in karst caves. A careful evaluation of pollen deposited in and outside the cave is the premise to do paleo-pollen studies, which not only important for past climate change studies, but also for archaeological studies in caves where archaeological remains preserved. After three round of review, I saw that previous reviewers had provided good suggestions and the authors had dealt with them carefully. And the manuscript has been improved a lot since the original one. I think the current manuscript is almost ready for publication. But please still try to consider my following suggestion:

I understand that the authors had thought about the influence of the structure of caves on the transportation and deposition of pollen. In fact, it is one of the reason why the authors did this study in these two caves. I would suggest to provide a map of the vertical profile of the sampled part of the cave to show the slope of the cave surface. It is important for the transportation of pollen and sediments in the cave, which also effects the explanation of the pollen results in this study. Therefore, the influence of geomorphology of the inside of the cave on the pollen results should be carefully considered.

**[Authors’ response]**

Thank you very much for your insightful comments. In response to your suggestion, we have incorporated field-measured cave-morphology data to generate vertical-profile maps of both Yinhegong Cave and Dongkou Cave, which are now presented as part of Figure 2.

The updated Figure 2 is provided below.

Once more, we thank you for your incisive remarks. Your observations were both pertinent and thought-provoking. After a thorough re-examination of the manuscript, we have integrated these suggestions and are confident that the resulting revisions have markedly strengthened the paper; we hope that these changes now satisfy your expectations.
